# Phase 3 Trial of a Small-volume Subcutaneous 6-Month Duration Leuprolide Acetate Treatment for Central Precocious Puberty

**DOI:** 10.1210/clinem/dgaa479

**Published:** 2020-08-01

**Authors:** Karen O Klein, Analía Freire, Mirta Graciela Gryngarten, Gad B Kletter, Matthew Benson, Bradley S Miller, Tala S Dajani, Erica A Eugster, Nelly Mauras

**Affiliations:** 1 Rady Children’s Hospital and University of California, San Diego, California; 2 Centro de Investigaciones Endocrinológicas “Dr. César Bergadá” (CEDIE) CONICET – FEI – División de Endocrinología, Hospital de Niños Ricardo Gutierrez, Buenos Aires, Argentina; 3 MultiCare Institute for Research and Innovation, Tacoma, Washington; 4 Nemours Children’s Hospital, Orlando, Florida; 5 University of Minnesota Masonic Children’s Hospital, Minneapolis, Minnesota; 6 School of Osteopathic Medicine in Arizona, A.T. Still University, Mesa, Arizona; 7 Riley Hospital for Children at Indiana University Health, Indianapolis, Indiana; 8 Nemours Children’s Health System, Jacksonville, Florida

**Keywords:** central precocious puberty, leuprolide acetate, gonadotropin releasing hormone agonists

## Abstract

**Context:**

Gonadotropin-releasing hormone agonists (GnRHas) are standard of care for central precocious puberty (CPP). A 6-month subcutaneous injection has recently been approved by the Food and Drug Administration.

**Objective:**

Determine efficacy, pharmacokinetics, and safety of 6-month 45-mg subcutaneous leuprolide acetate for CPP.

**Design:**

Phase 3 multicenter, open-label, single-arm study.

**Setting:**

25 sites in 6 countries.

**Subjects:**

64 GnRHa-naïve children with CPP (age: 7.5 ± 0.1 years) received study drug: 59 completed the study.

**Intervention(s):**

2 doses of 45-mg subcutaneous leuprolide acetate (0.375 mL) at 0 and 24 weeks; children were followed for 48 weeks.

**Main Outcome Measure(s):**

Percentage of children with serum luteinizing hormone (LH) <4 IU/L 30 minutes following GnRHa stimulation at week 24.

**Results:**

54/62 (87%) children achieved poststimulation LH <4 IU/L at week 24; 49/56 (88%) girls and 1/2 boys maintained peak LH <4 IU/L at week 48. Mean growth velocity decreased from 8.9 cm/year at week 4 to 6.0 cm/year at week 48. Mean bone age was advanced 3.0 years beyond chronological age at screening and 2.7 years at week 48. Breast pubertal stage regressed or was stable in 97% of girls and external genitalia development regressed in both boys. Adverse events were mild and did not cause treatment discontinuation.

**Conclusions:**

A small volume of 45-mg subcutaneous leuprolide acetate administered at a 6-month interval effectively suppressed pubertal hormones and stopped or caused regression of pubertal progression. This long-acting GnRHa preparation of leuprolide acetate is a new, effective, and well-tolerated therapy for children with CPP.

GnRH agonists (GnRHas) are considered standard treatment for children with central precocious puberty (CPP) (1-3). Optimal treatment inhibits the physical signs of pubertal progression, returns growth velocity to normal prepubertal rates, decreases the rate of bone maturation, improves predicted adult height, and suppresses pubertal hormonal measures (4-7).

Currently available treatments for CPP in the United States include intramuscular leuprolide acetate injections, intramuscular triptorelin injections, and subcutaneous histrelin acetate implants (8-10). These options have differing routes of administration, dosing volumes and duration of action. For example, intramuscular injections of leuprolide acetate have volumes of 1 or 1.5 mL and are administered every 1 or 3 months (8). Triptorelin has a volume of 2 mL and is administered intramuscularly every 24 weeks (9). The histrelin implant is inserted every year and may be effective for up to 2 years (10, 11). Insertion and removal of implants may require anesthesia (often local, sometimes general) (2, 10), and they may be left in situ for longer than intended if the patient is lost to follow-up (11, 12). Commonly reported local adverse events (bruising, pain, injection reactions, and sterile abscesses) are likely associated with methods of administration and the excipients necessary to prolong release of active drug and clinical effect (13-17). Selection of therapy is usually individualized according to patient needs and preferences.

When first used as therapy, GnRHas were administered by daily subcutaneous injection (18). An international consortium of pediatric endocrinology society representatives recently identified a preference for long-acting subcutaneous injections over the intramuscular route as an example of an improvement in clinical care, by lowering injection site trauma (2).

This Phase 3 study was designed to evaluate the efficacy, pharmacokinetics (PK) and safety of 6-month 45-mg subcutaneous leuprolide acetate (Fensolvi^®^) in children with CPP. The unique formulation uses an innovative proprietary polymeric gel extended-release technology that enables sustained and consistent release of leuprolide over a 6-month dosing period, with the benefits of subcutaneous injection, small injection volume (0.375 mL) and a short needle (5/8-inch, 18-gauge). It was approved by the Food and Drug Administration on May 1, 2020 (19).

## Materials and Methods

### Study design

The study was designed as a multicenter, open-label, single-arm, adaptive Phase 3 protocol and was conducted between August 2015 and September 2018 (NCT02452931). All study documents were approved by an Institutional Review Board or Independent Ethics Committee for each site prior to initiation of the study. All aspects of the study were conducted in accordance with International Council on Harmonisation Good Clinical Practice principles. Caregivers gave written informed consent and children gave assent, when applicable.

A 2-part adaptive protocol design was used as the study drug had not previously been administered to children. Part A evaluated the efficacy, tolerability, and safety of 2 doses of 45-mg subcutaneous leuprolide acetate administered at a 24-week interval. The intervention was considered effective if luteinizing hormone (LH) was suppressed to <4 IU/L at week 24 (month 6) in response to an abbreviated GnRHa stimulation test. Part B was a contingency to evaluate a shorter 5-month interval if a planned interim analysis demonstrated that the study drug did not effectively suppress LH levels for 24 weeks in at least 80% of the children. Following the interim analysis of a subset of children studied in Part A at 24 weeks following the first dose, review by an independent Drug Safety Monitoring Board determined that initiation of Part B was not necessary.

### Study population

Target recruitment was a minimum of 60 children. Girls aged 2 to 8 years and boys aged 2 to 9 (both inclusive) with a confirmed diagnosis of CPP and naïve to GnRHa treatment were eligible for inclusion. Criteria for diagnosis of CPP included (1) clinical evidence of puberty, defined as pubertal stage ≥2 for breast development in girls or testicular volume ≥4 mL in boys; (2) advanced bone age (BA) by at least 1 year compared with chronological age (CA); and (3) a pubertal LH response to >5 mIU/L at 30 minutes subsequent to a GnRHa stimulation test. Key exclusion criteria included gonadotropin-independent (peripheral) precocious puberty; nonprogressing isolated premature thelarche; previous seizures; and any condition, chronic illness, or treatment that, in the opinion of the investigator, could interfere with growth or other study endpoints (eg, chronic steroid use not including mild topical steroids, renal failure, diabetes mellitus, moderate to severe scoliosis, and previously treated intracranial tumor).

### Study treatment

Eligible children received 2 single doses of 45-mg subcutaneous leuprolide acetate at a 24-week interval and were evaluated over the 48-week study period. The first injection was administered at baseline (day 0) and the second at week 24. End of treatment was defined as week 48. Subcutaneous leuprolide acetate (45 mg) was prefilled in 2 separate, sterile syringes. Syringe A contained a viscous lactide–glycolide copolymer delivery system and Syringe B contained the lyophilized leuprolide acetate powder. The 2 syringes were joined and the leuprolide acetate powder was reconstituted with the polymeric gel delivery system by mixing for 45 seconds until a uniform suspension was obtained. The suspension (0.375 mL injection volume) was drawn into Syringe B and injected subcutaneously into the abdominal area through a 5/8-inch, 18-gauge safety needle. Injections had to be administered within a 30-minute window following reconstitution. Investigators had the option to use topical or local anesthetics to “numb” the injection site and record as concomitant medication.

### Study assessments

GnRHa stimulation tests were performed by subcutaneous administration of 20 μg/kg or 500 μg (fixed dose) aqueous leuprolide acetate. Forty-nine children from 22 sites underwent tests using 20 μg/kg dosing with a mean dose administered of 755 μg. Fixed dose tests were used at the other 3 sites. Both test approaches have been well characterized as appropriate and clinically qualified, and no differences were observed in results from children receiving one or the other (20). Assessments of serum LH, follicle-stimulating hormone (FSH), and estradiol (E_2_) or testosterone (T) levels were performed at 0 and 30 minutes poststimulation. Tests for diagnosis were performed at screening, and at weeks 12, 24, 36, and 48 to assess gonadotropin suppression during treatment. Serum concentrations of LH ≥5 IU/L at 30 minutes were considered diagnostic for CPP at screening, and effective LH suppression to prepubertal levels on treatment was defined as LH <4 IU/L at 30 minutes. LH levels 30 minutes after GnRH stimulation are referred to as peak LH throughout the text. LH levels at time 0 or at a visit where a stimulation test was not performed are referred to as random LH.

Blood samples for random LH, FSH, and E_2_ or T were also collected at weeks 4, 20, and 44. Serum LH and FSH were both assessed using a validated central Cobas ECLIA assay (Roche Diagnostics GmbH, Sandhofer Strasse 116, D-68305 Mannheim, Germany) with lower limits of detection (LLOD) of 0.100 IU/L (The Doctors Laboratory Ltd, UK). E_2_ samples were batched and analyzed by liquid chromatography tandem mass spectrometry/mass spectrometry (LC-MS/MS) (ABS Laboratories Ltd, UK). The lower limit of quantitation (LLOQ) for the highly specific E_2_ assay was 10 pg/mL. Testosterone levels were measured using a validated chemiluminescent microparticle immunoassay with an LLOD of 11.5 ng/dL (The Doctors Laboratory Ltd, UK).

Anthropometric and radiographic data including height by wall-mounted stadiometer (screening, baseline, weeks 4, 12, 20, 24, 36, 44, and 48), BA (screening, weeks 24 and 48), and pubertal staging (screening, weeks 12, 24, 36, and 48) were collected. Growth velocity was calculated using height measurements taken at baseline, weeks 4, 12, 24, 36, and 48. Growth velocity at week 4 was used as a substitute for a baseline value for measurement consistency, acknowledging the very short interval of 4 weeks. Radiographs of the left hand and wrist were used to determine BA using the Greulich and Pyle method (21, 22). A qualified provider at each site determined study eligibility during screening, and BA was assessed at subsequent study visits by a central reader (Intrinsic Imaging, LLC, USA) who was blinded to treatment timing or dose. Rate of advancement in BA was determined by differences between BA and CA (BA-CA) at each measured timepoint. Pubertal maturation was categorized with a modified Tanner staging system using breast development as assessed by palpation (girls), external genitalia (boys), and pubic hair (both sexes) (23).

To evaluate the PK of the study drug, blood samples for analysis of leuprolide concentrations were collected at screening, 1, 4, and 6 hours after the first dose of the study drug, and also at weeks 4, 12, 20, 24, 36, 44, and 48. All samples were processed by a central laboratory (Syneos Health [formerly inVentiv Health Clinique Inc.], Canada). A validated bioanalytical LC-MS/MS method with an LLOD of 0.025 ng/mL was used to measure serum leuprolide concentrations.

### Statistical analyses

Descriptive statistics were calculated for data collected at the specified timepoints. Continuous data were summarized using mean, standard error (SE), median, minimum, maximum, and number of children. Categorical data were summarized using frequencies and proportions.

The proportions of children demonstrating suppression of LH and other sex steroid hormones to prepubertal levels were based on the total number of children with data available at each respective timepoint.

Other analyses included change in growth velocity, BA-CA, ratio of BA to CA, change in pubertal stage from screening to end-of-study, change in body mass index (BMI), and change in hormone concentration from baseline (LH, FSH, and E_2_ or T). Growth velocity was calculated using the difference in height between visits and annualized for presentation. Effective LH suppression to prepubertal levels was defined as LH <4 IU/L based on standards used for other approved GnRHa agents (24-26). Effective suppression of sex steroids to prepubertal levels was defined as E_2_ <20 pg/mL for girls and T <28.4 ng/dL for boys, and the criterion for effective FSH suppression was a level of <2.5 IU/L (27). Values below the LLOD/LLOQ were entered as the LLOD/LLOQ for analyses. Paired t-tests were used for comparisons of BMI between timepoints.

Statistical analysis of safety was descriptive only. Treatment-emergent adverse events (TEAEs) were defined as any adverse events (AEs) occurring or worsening on or after the first dose of study drug.

## Results

**Figure 1.**
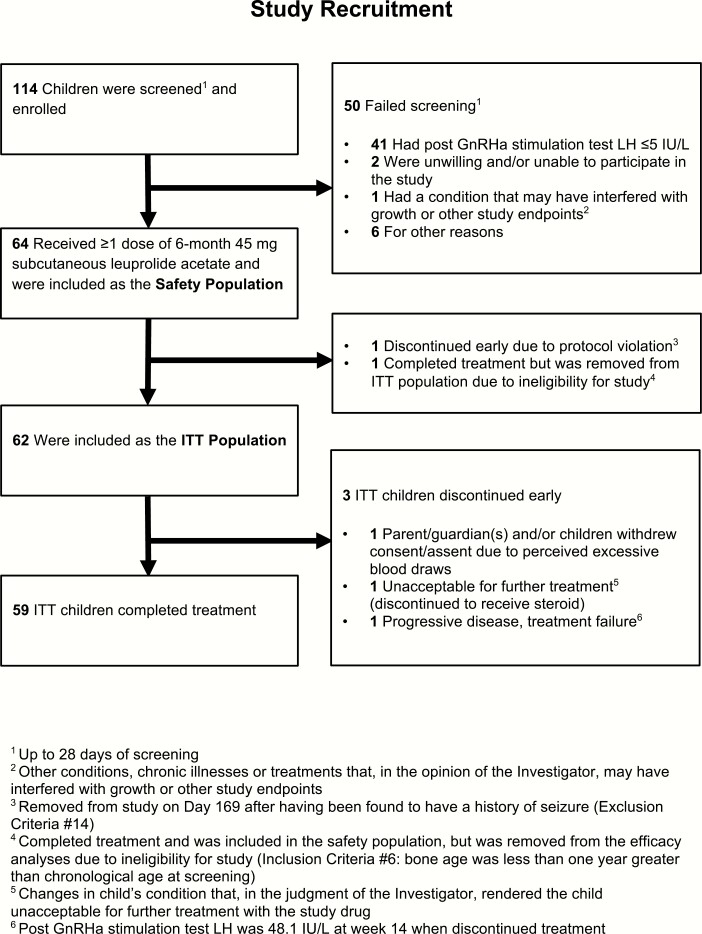
Study recruitment. ^1^Up to 28 days of screening. ^2^Other conditions, chronic illnesses or treatments that, in the opinion of the investigator, may have interfered with growth or other study endpoints. ^3^Removed from study on day 169 after having been found to have a history of seizure (exclusion criteria #14). ^4^Completed treatment and was included in the safety population, but was removed from the efficacy analyses due to ineligibility for study (inclusion criteria #6: bone age was less than 1 year greater than chronological age at screening). ^5^Changes in child’s condition that, in the judgment of the investigator, rendered the child unacceptable for further treatment with the study drug. ^6^Post-GnRHa stimulation test LH was 48.1 IU/L at week 14 when discontinued treatment.

Sixty-four children were screened and received at least 1 dose of 45-mg subcutaneous leuprolide acetate and were included in the safety population (Fig. 1). Causes of CPP (idiopathic or organic) were not captured in study documents at screening, but children with an unstable intracranial tumor (or an intracranial tumor requiring surgery or cerebral irradiation) were excluded by the provider prior to screening. One child was discontinued early and another was found to be ineligible for the study after treatment, leaving 62 who fulfilled the protocol eligibility criteria and were designated as the Intent-to-Treat (ITT) population. Mean age at baseline in the ITT population was 7.5 ± 0.1 years (girls 7.4 ± 0.1 years [range: 4-8]; both boys 9 years) and 60/62 (96.8%) were girls (Table 1). Approximately half were Caucasian (32/62; 51.6%), with the remainder being African American or other ethnic minorities. Three children in the ITT population discontinued treatment early and the remaining 59 children (57 girls, 2 boys) completed the study. One child withdrew due to perceived excessive blood draws, 1 required additional steroid treatment based on the judgment of the investigator due to changes in condition (no alternate diagnosis provided), and 1 had continued progression of puberty with a peak LH of 48.1 IU/L at week 14.

**Table 1.** Baseline demographics and characteristics (intent-to-treat population)

Variable		N = 62	
Age, years	Mean ± SE	7.5 ± 0.1	
	Min, max	4, 9	
Sex, n (%)	Boys	2	(3.2)
	Girls	60	(96.8)
Ethnicity, n (%)	Caucasian	32	(51.6)
	African American	15	(24.2)
	American Indian or Alaska native	5	(8.1)
	Asian	3	(4.8)
	Native Hawaiian or other Pacific Islander	1	(1.6)
	Unwilling to provide	1	(1.6)
	Other	5	(8.1)
Height, cm	Mean ± SE	136.6 ± 1.0	
	Median (min, max)	136.6 (109.0, 153.0)	
Growth velocity^*a*^, cm/year	Mean ± SE	8.9 ± 1.7	
	Median	8.8	
BMI, kg/m^2^	Mean ± SE	18.5 ± 0.4	
	Median (min, max)	18.2 (13.9, 25.3)	
BA-CA^*b*^, year	Mean (SE)	3.0 ± 0.1	
	Median (min, max)	3.2 (0.8, 5.8)	
Pubertal staging^*c*^, n (%)	Stage 2	5	(8)
	Stage 3	45	(73)
	Stage 4	10	(16)
	Stage 5	2	(3)

Abbreviations: BMI, body mass index; SE, standard error.

^*a*^Earliest growth velocity data at week 4.

^*b*^Difference between bone age and chronological age at time of measurement.

^*c*^N = 60 for breast development, girls; N = 2 for development of external genitalia, boys; both boys are at stage 3 at baseline.

### Serum hormone concentrations

**Figure 2.**
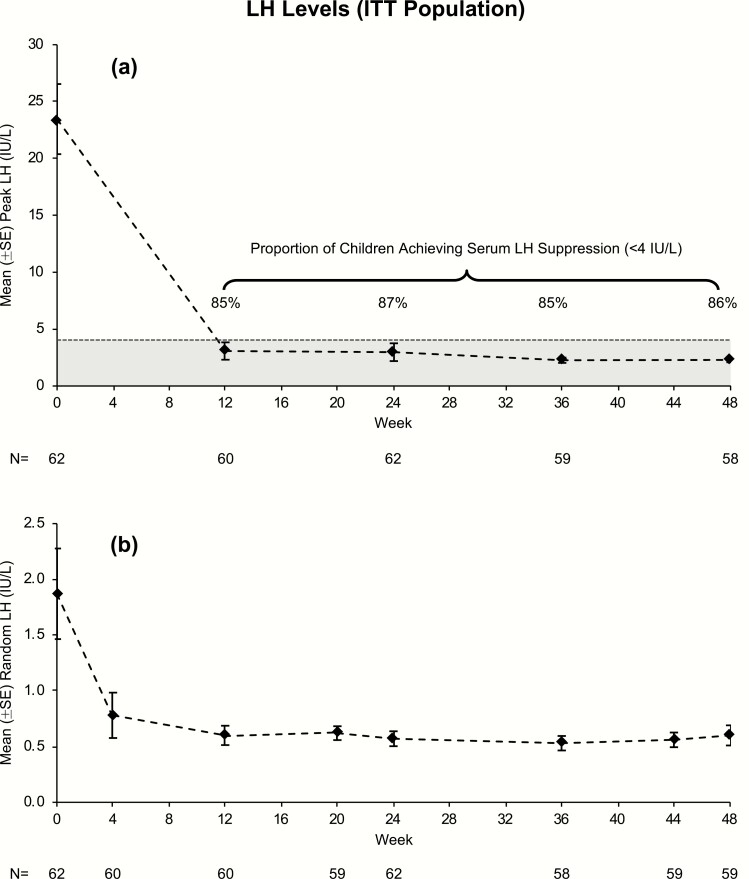
(A) Mean (±SE) peak LH level and proportion of children who achieved peak LH < 4 IU/L (ITT population). (B) Mean (±SE) random LH (IU/L) (ITT population). ^1^Screening random (pre-GnRHa stimulation test) LH data.

In the ITT population, 87% (54/62) of children (52/60 girls) demonstrated suppression of peak LH to <4 IU/L at week 24 (Table 2). LH levels of <4 IU/L were achieved by ≥85% of children at each timepoint (Fig. 2). Forty-nine of 56 (88%) girls and 1 of 2 boys maintained suppression of peak LH <4 IU/L at week 48. Mean peak LH levels declined from 23.5 ± 3.1 IU/L (range: 5.1-112.8) at screening to 3.0 ± 0.8 IU/L (range: 0.3-48.1) at week 24 and 2.3 ± 0.2 IU/L (range: 0.3-8.0) at week 48 (Table 3). Mean random LH levels were ≤0.6 IU/L and ranged from 0.1 to 3.9 IU/L across all timepoints after week 12.

**Table 2.** Proportion of children achieving serum hormone suppression (intent-to-treat population)

Endpoint target^*a*^	Proportion of children achieving endpoints, % (n/N)			
	**Week 12**	**Week 24**	**Week 36**	**Week 48**
LH < 4 IU/L	85 (51/60)	87 (54/62)^*b*^	85 (50/59)	86 (50/58)
Estradiol < 20 pg/mL	98 (56/57)	97 (58/60)	98 (56/57)	98 (55/56)
Testosterone < 28.4 ng/dL	100 (2/2)	100 (2/2)	100 (2/2)	50 (1/2)
FSH < 2.5 IU/L	62 (37/60)	66 (41/62)	44 (26/59)	55 (32/58)

Abbreviations: GnRH, gonadotropin-releasing hormone; FSH, follicle-stimulating hormone; LH, luteinizing hormone.

^*a*^Post GnRH agonist stimulation.

^*b*^Primary efficacy endpoint.

**Table 3.** Serum hormone concentrations (intent-to-treat population)

Study visit	Sample time^*a*^	Luteinizing hormone		Estradiol^*b*^		Testosterone^*b*^		Follicle-stimulating hormone	
		**Mean** ± **SE (range)** **(IU/L)**	**N**	**Mean** ± **SE (range)** **(pg/mL)**	**N**	**Mean** ± **SE (range)** **(ng/dL)**	**N**	**Mean** ± **SE (range)** **(IU/L)**	**N**
Screening	Pre-stim	1.9 ± 0.4 (0.1, 22.0)	62	27.3 ± 3.3 (10.0, 117.1)	57	119.7 ± 40.3 (80.8, 158.6)	2	4.1 ± 0.3 (0.8, 16.9)	62
	Post-stim	23.5 ± 3.1 (5.1, 112.8)	62	25.6 ± 2.9 (10.0, 120.9)	59	112.5 ± 49.0 (63.5, 161.5)	2	11.0 ± 1.0 (1.1, 52.6)	62
Visit 1 (baseline)	Baseline	3.5 ± 1.2 (0.1, 68.9)	62	25.3 ± 3.3 (10.0, 119.0)	60	285.5 ± 40.4 (245.2, 325.9)	2	3.9 ± 0.3 (0.5, 11.2)	62
Visit 2 (week 4)		0.8 ± 0.2 (0.1, 12.2)	60	14.6 ± 3.3 (10.0, 190.1)	58	24.5 ± 13.0 (11.5, 37.5)	2	1.0 ± 0.1 (0.2, 5.5)	60
Visit 3 (week 12)	Pre-stim	0.6 ± 0.1 (0.1, 3.9)	60	10.6 ± 0.3 (10.0, 19.1)	57	15.9 ± 4.3 (11.5, 20.2)	2	1.5 ± 0.1 (0.2, 5.0)	60
	Post-stim	3.1 ± 0.8 (0.1, 46.7)	60	10.8 ± 0.6 (10.0, 42.8)	57	15.9 ± 4.3 (11.5, 20.2)	2	2.8 ± 0.3 (0.2, 12.5)	60
Visit 4 (week 20)		0.6 ± 0.1 (0.1, 2.1)	59	10.5 ± 0.3 (10.0, 23.2)	57	23.1 ± 11.5 (11.5, 34.6)	2	1.4 ± 0.1 (0.2, 5.1)	59
Visit 5 (week 24)	Pre-stim	0.6 ± 0.1 (0.1, 3.3)	62	10.3 ± 0.2 (10.0, 21.5)	59	18.8 ± 7.2 (11.5, 26.0)	2	1.2 ± 0.1 (0.2, 3.3)	62
	Post-stim	3.0 ± 0.8 (0.3, 48.1)	62	10.6 ± 0.3 (10.0, 24.5)	60	15.9 ± 4.3 (11.5, 20.2)	2	2.4 ± 0.2 (0.2, 9.2)	62
Visit 6 (week 36)	Pre-stim	0.5 ± 0.1 (0.1, 2.9)	58	10.2 ± 0.2 (10.0, 20.9)	56	11.5 ± 0.0 (11.5, 11.5)	2	1.4 ± 0.1 (0.1, 3.5)	58
	Post-stim	2.3 ± 0.2 (0.3, 10.1)	59	10.4 ± 0.2 (10.0, 19.9)	57	11.5 ± 0.0 (11.5, 11.5)	2	3.1 ± 0.3 (0.2, 9.6)	59
Visit 7 (week 44)		0.6 ± 0.1 (0.1, 2.5)	59	11.0 ± 0.4 (10.0, 23.4)	57	11.5 ± 0.0 (11.5, 11.5)	2	1.4 ± 0.1 (0.1, 4.0)	59
End of treatment (week 48)	Pre-stim	0.6 ± 0.1 (0.1, 3.3)	59	10.1 ± 0.1 (10.0, 15.5)	57	17.3 ± 5.8 (11.5, 23.1)	2	1.4 ± 0.1 (0.1, 5.4)	59
	Post-stim	2.3 ± 0.2 (0.3, 8.0)	58	10.5 ± 0.3 (10.0, 22.9)	56	27.4 ± 15.9 (11.5, 43.3)	2	3.0 ± 0.3 (0.1, 13.1)	58

^*a*^ Pre-stim, prestimulation test; post-stim, poststimulation test.

^*b*^No expected change poststimulation at time point measured.

Eight girls did not achieve LH suppression to <4 IU/L at week 24. One girl (with E_2_ 48.9 pg/mL at screening) discontinued the study early at week 14 due to progressive disease; she escaped suppression by week 14 and her data at that timepoint (peak LH 48.1 IU/L and E_2_ 24.6 pg/mL) were applied to the next available visit (week 24). Among the other 7 girls who completed the study, 4 had peak LH levels between 4.2 and 4.8 IU/L at week 24, with similar values at week 48 (range: 4.1-6.1). The other 3 had higher peak LH levels at week 24 (5.5, 5.8, and 14.2 IU/L), but all 3 had peak LH <4 IU/L by week 48. Two of the 7 girls had E_2_ >20 pg/mL at screening, 6 of them achieved E_2_ suppression to <10 pg/mL (one was 15.2 pg/mL) at week 24, and all 7 had E_2_ <10 pg/mL at week 48. Note that among children with peak LH <4 IU/L at week 24, 4 of them had a slightly higher peak LH at week 48 (4.0, 4.2, 4.2, and 4.7 IU/L). Pubertal staging for breast development regressed or was stable for 6 girls from baseline to week 24, and for all 7 girls from baseline to week 48 (3 girls regressed 2 stages, 2 regressed 1 stage, and 2 were stable).

Levels of sex hormones at each visit are shown in Table 3. All but 2 children achieved suppression of E_2_ <20 pg/mL (58/60; 97%) or T <28.4 ng/dL (2/2; 100%) at week 24, meeting predefined prepubertal targets (Table 2). As girls starting puberty often have E_2_ levels between 10 and 20 pg/mL, we also present the E_2_ data using a cutoff of 10 pg/mL. 90% (54/60) of girls at week 24 and 98% (55/56) at week 48 achieved E_2_ <10 pg/mL. Ninety-two percent (212/230) of all E_2_ measurements at and after week 12 were <10 pg/mL. Both boys had T levels reduced to <28.4 ng/dL at weeks 12, 24, and 36. At week 48, 1 boy’s T level remained suppressed below this level; however, the other boy had an above-target T of 43.2 ng/dL. Interestingly, this boy’s other assessments (decrease in BA-CA from 3.25 years at screening to 2.83 years at week 48, and no progression in pubertal staging) were consistent with effective treatment. Of note, his LA levels were measurable through week 44 but undetectable at week 48. A majority of children (41/62, 66%) met the criterion for suppression of FSH (<2.5 IU/L) at week 24 and 55% of children achieved this at week 48.

### Growth velocity, bone age advancement, pubertal staging, and BMI

**Figure 3.**
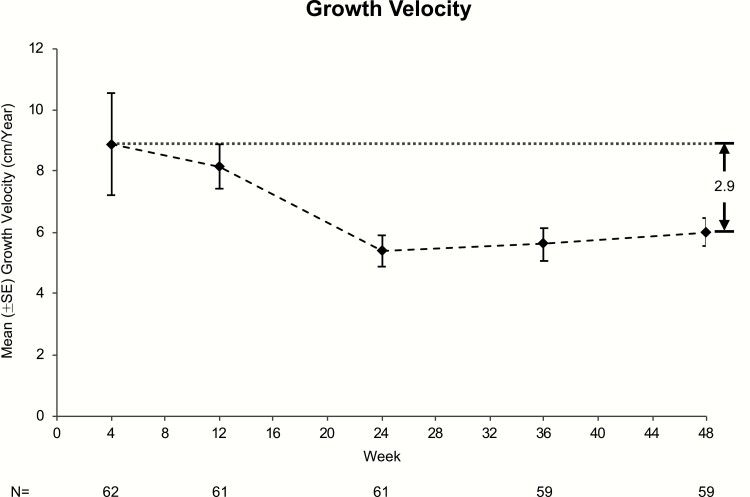
Mean (±SE) growth velocity over time (ITT population). ^1^Baseline defined as the last nonmissing assessment done prior to or on the date of first injection.

A little more than half of the children experienced a decrease in growth velocity by week 24 (35/61, 57.4%) and week 48 (31/59, 52.5%). Mean growth velocity slowed from 8.9 ± 1.7 cm/year at week 4 to 5.4 ± 0.5 cm/year at week 24 and 6.0 ± 0.5 cm/year at week 48 (Fig. 3). Median growth velocity showed a similar trend (week 4: 8.8 cm/year, week 24: 4.8 cm/year, week 48: 5.7 cm/year).

Slowing in the rate of advancement in BA continued throughout the 48-week study period. Mean values for BA-CA decreased significantly from 3.0 ± 0.1 years at screening to 2.8 ± 0.1 years at week 24 (*P* = .001), and 2.7 ± 0.1 years at week 48 (*P* < .001). Analyses for the ratio of BA to CA were consistent with those for BA-CA (data not shown).

Clinical signs of puberty stabilized or regressed in almost all girls (55/57). Regression in pubertal stage for breast development was observed in 46% (26/57) of girls by week 48, and stabilization of breast stage was seen in another 51% (29/57). At week 48 both boys had regressed from stage 3 to stage 2 in appearance of external genitalia. Pubic hair development remained unchanged from screening for 71% of children and had regressed in 10% by week 48.

Mean BMI at baseline was 18.2 ± 0.4 kg/m^2^ (median: 18.6; range: 13.9-25.3). BMI increased slightly from baseline to week 24 (18.8 ± 0.4 kg/m^2^; median: 19.1; range: 12.9-27.2; *P* < .01) but did not significantly increase between week 24 and week 48 (19.1 ± 0.4 kg/m^2^; median: 19.7; range: 13.4 to 27.4; *P* = .16).

### Pharmacokinetic analysis of leuprolide

**Figure 4.**
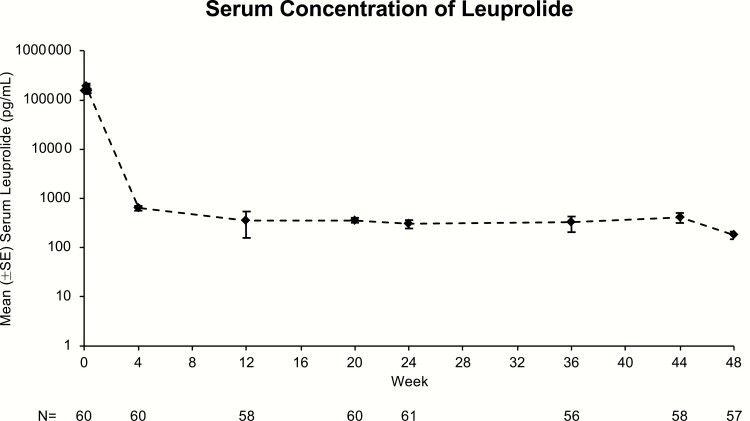
Mean (±SE) serum concentration of leuprolide over 48 weeks (ITT population).

A characteristic initial burst release of serum leuprolide was observed within 1 to 6 hours of dosing; however, this represented only a small proportion of total exposure. Leuprolide levels peaked 4 hours after injection (mean C_max_: 212.3 ng/mL), and the average observed concentration following the burst through week 24 was 10.9 ± 1.6 ng/mL (range: 0.1-82.2) overall (Table 4). Mean serum leuprolide concentration decreased to 0.63 ng/mL at week 4 and remained stable from week 12 to week 44. No accumulation of leuprolide was observed following the second injection. In the plateau phase (4-48 weeks), mean leuprolide level was 0.37 ng/mL with a range of 0.18 ng/mL to 0.63 ng/mL (Fig. 4).

**Table 4.** Pharmacokinetics of leuprolide

Burst phase			Plateau phase	Overall
**C**_**max**_ **(ng/mL)**	**T**_**max**_^***a***^ **(hours)**	**AUC** ^*b*^ (ng*day/ mL)	**AUC** ^***b***^ (ng*day/ mL)	**C**_**avg**_^***c***^ **(ng/mL)**
212.3	3.7	39.1	1760.7	10.9

^*a*^Time at which the C_**max**_ is observed.

^*b*^The area under the plasma drug concentration–time curve.

^*c*^Area under the plasma drug concentration–time curve from day 7 to day 169 divided by 162 days.

### Safety

Injections of 45-mg subcutaneous leuprolide acetate administered every 24 weeks were well tolerated. Local anesthetics, primarily lidocaine, were administered with 82% of the injections. AEs did not result in withdrawal of any child from the study or discontinuation of study drug. Two serious AEs (wheezing and rash) that were considered unrelated to the study drug were reported for 1 child.

TEAEs reported in ≥5% of the safety population included injection site pain (31%), nasopharyngitis (22%), pyrexia (17%), headache (16%), and cough (13%) (Table 5). All instances of injection site pain were mild (Grade 1). Other adverse reactions included emotional disorder (2%) and irritability (2%). Thirty-four percent of children experienced treatment-related AEs. No cases of sterile abscess were reported. “Injection-related” AEs included events related both to injections of study drug and to leuprolide used for stimulation tests; it was not possible to separate causality.

**Table 5.** Summary of TEAEs occurring in ≥5% of children (safety population)

System organ class preferred term^*a*^	Safety population (N = 64) n (%)
Any TEAE^*b*^	53 (83)
Any treatment-related adverse event	22 (34)
Injection site pain	20 (31)
Nasopharyngitis	14 (22)
Pyrexia	11 (17)
Headache	10 (16)
Cough	8 (13)
Abdominal pain	6 (9)
Injection site erythema	6 (9)
Nausea	5 (8)
Constipation	4 (6)
Vomiting	4 (6)
Upper respiratory tract infection	4 (6)
Bronchospasm	4 (6)
Productive cough	4 (6)
Hot flush	3 (5)

Abbreviations: AE, adverse event; TEAE, treatment-emergent adverse event.

^*a*^ Children with 2 or more adverse events in the same system organ class (or within the same preferred term) were counted only once for that system organ class (or preferred term).

^*b*^A TEAE was defined as any AE occurring or worsening on or after the first dose of study drug.

## Discussion

The most important treatment objectives in children with CPP are suppression of pubertal hormones, halting of progression of secondary signs of sexual maturation, and preservation of adult height potential by slowing the rate of bone maturation. Secondary goals center on reduction of adverse psychosocial consequences by improving alignment between physical development, CA, and emotional maturity (28, 29). However, psychosocial outcomes were not assessed in the present study. Although GnRHa therapies are proven effective, alternatives in route of administration, injection volume, and treatment frequency may improve patient experience and increase adherence to treatment protocols. An analysis of children treated with leuprolide acetate for up to 8 years determined that over 74% of them did not receive injections within the recommended administration period (30). Nonadherence to dosing schedules may be associated with suboptimal outcomes (31). Thus, the availability of effective, long-acting, safe, and conveniently administered treatments for CPP would be an important advance.

This Phase 3 study evaluated the efficacy, PK and safety of 6-month 45-mg subcutaneous leuprolide acetate in children with CPP. This is the first study to investigate a 6-month, small-volume GnRHas administered via subcutaneous injection in this patient population. Results suggest that the study drug represents a promising novel treatment option that may address some unmet needs.

Six-month 45-mg subcutaneous leuprolide acetate suppressed peak LH and gonadal sex steroids to prepubertal levels in >85% of the children at all study assessments and, therefore, may be considered effective for the treatment of children with CPP. Although 8 children did not achieve the primary study endpoint (peak LH of <4 IU/L at 24 weeks), almost all of them had improvements in other assessments, suggesting that their CPP was being adequately treated. By week 24, most children achieved prepubertal levels of E_2_ (97%) or T (100%). However, sex steroid levels should be interpreted with caution as they were obtained at random timepoints in this study and were not measured by an ultrasensitive assay. Since the sensitivity of the E_2_ assay was 10 pg/mL, and all values below this level were conservatively entered as 10 pg/mL, actual mean E_2_ levels on treatment were likely lower than those presented.

Additionally, 6-month 45-mg subcutaneous leuprolide acetate decelerated growth velocity and slowed advancement in BA. Although regression or stabilization in pubertal staging for breast development was seen in the vast majority of girls, 3 had a stage 3 assessment at week 48. This finding is not unusual, as the firmness of breast tissue was not documented and there may have been persistence of fat tissue despite reductions in glandular tissue. The relative lack of regression in pubic hair development was not unexpected as growth of pubic hair is primarily influenced by release of sex steroids from the adrenal glands and pubic hair persists after hormone withdrawal. The longer growth cycle for pubic hair makes regression difficult to observe (32), even over a 48-week study period. The effect of GnRHa treatment on BMI varies between studies in children with CPP (33, 34). The present study showed a small but statistically significant increase in BMI during the first 24 weeks of treatment, but no further significant increase by week 48. Although cross-study comparisons should be treated with caution, the overall efficacy and safety of subcutaneous leuprolide acetate appears similar to that of other approved GnRHa used for treating CPP (24-26, 35-38).

Another objective of using the subcutaneous route of administration for a GnRHa is to lower the risk of injection site reactions, including secondary swelling, intramuscular hematoma, and bone or nerve injury (2, 13, 39, 40). Subcutaneous injections avoid scarring and risk of injury and infection that may occur following placement of some subdermal implants (41, 42). Research suggests that the convenience and tolerability of subcutaneous injection will likely be especially valuable for children (14, 43, 44), and this route of administration has been supported by members of multiple international pediatric endocrinology societies (2). Subcutaneous leuprolide acetate also has flexible timing of administration with a 30-minute window following reconstitution; the window is 2 hours for intramuscular leuprolide acetate, and triptorelin requires immediate injection after reconstitution (8, 9, 19). Based on the occurrence of AEs, vital signs, and laboratory results seen in the study, the overall safety profile of 45-mg subcutaneous leuprolide acetate appears similar to other GnRHa (24-26, 36-38). However, the study design did not include direct comparisons of intramuscular and subcutaneous routes of injection. AEs such as convulsions and sterile abscesses have been observed in children receiving GnRHa, but they were not observed in the current study, possibly as only approximately 120 injections were evaluated.

Subcutaneous leuprolide acetate has been used to treat men with advanced prostate cancer for more than 15 years (45). The PK and efficacy data from the current study in children with CPP provide further evidence of the consistent and sustained delivery of leuprolide acetate using a polymeric gel extended-release technology over the dosing period.

There are a number of limitations to this study. There were many screen failures, likely as peak LH was measured only 30 minutes after injection; many responses may have been missed as the peak may occur more than 1 hour after injection (46). The screen failures were also likely to be children who had not yet robustly entered into puberty, and perhaps had slower progression of puberty. The present study’s more stringent inclusion criteria were unlikely to have affected the efficacy and safety results. The absence of a control group was unavoidable as use of placebo would have been unethical in a pediatric population where effective treatments for CPP are available. Another limitation was that the study only included 2 boys, both of whom did not undergo assessment of testicular volume, and 1 who had no measurable concentration of leuprolide at 48 weeks. The ratio of boys to girls in the study was low; however, it was comparable to the general CPP population and no major differences in efficacy have been reported between the sexes in studies evaluating other GnRHa. Additionally, causes of CPP were not captured; however, as children with unstable intracranial tumors were excluded at screening and the study only included 2 boys, it is likely a high proportion of enrolled children had idiopathic CPP. An important limitation was that the LLOQ for the LC-MS/MS E_2_ assay was only 10 pg/mL and, therefore, levels <10 pg/mL were not accurately captured. Development of sterile abscess has been a concern when using GnRHa for CPP (17, 47). Although an 18-gauge needle was required in this study to inject the suspension due to its viscosity, no sterile abscesses were observed. Additionally, sterile abscess is not reported as an AE in the label for a product that uses the same formulation and needle size (45). Finally, the children were only evaluated for 48 weeks of treatment and additional data will be required for confirmation of long-term efficacy and safety.

The 45-mg, subcutaneous formulation of leuprolide acetate effectively reduced LH and sex steroids to prepubertal levels, regressed or stabilized clinical signs of puberty, reduced mean growth velocity, slowed the rate of advancement of bone maturation, and demonstrated a good safety profile. Approved by the Food and Drug Administration on May 1, 2020, it is the first leuprolide acetate therapy with a polymeric gel delivery system and a small injection volume administered subcutaneously every 6 months, and represents an effective, safe, and convenient addition to existing treatment options for children with CPP.

## Data Availability

Restrictions apply to the availability of data generated or analyzed during this study to preserve patient confidentiality or because they were used under license. The corresponding author will on request detail the restrictions and any conditions under which access to some data may be provided.

## Abbreviations

AE: adverse event
BA: bone age
BMI: body mass index
CA: chronological age
CPP: central precocious puberty
E2: estradiol
FSH: follicle-stimulating hormone
GnRHa: gonadotropin-releasing hormone agonist
ITT: intent to treat
LC-MS/MS: liquid chromatography tandem mass spectrometry/mass spectrometry
LH: luteinizing hormone
LLOD: lower limits of detection
LLOQ: lower limit of quantitation
T: testosterone
TEAE: Treatment-emergent adverse event

## Additional Information

***Disclosure Summary***: All authors were principal investigators of the trial funded by Tolmar Inc. but were not compensated for their contributions to this manuscript. Dr. Klein is a consultant for AbbVie, Arbor Pharmaceuticals, and Tolmar Pharmaceuticals. Dr. Benson is a consultant for Arbor Pharmaceuticals and Tolmar Pharmaceuticals. Dr. Miller is a consultant for Abbvie, Ascendis, BioMarin, Bluebird Bio, GeneScience, Novo Nordisk, Pfizer, Sandoz, Sanofi Genzyme, and Tolmar Pharmaceuticals, and has received research support from Alexion, Abbvie, Amgen, Ascendis, BioMarin, Novo Nordisk, Opko, Protalix, Sandoz, Sangamo, Sanofi Genzyme, Tolmar Pharmaceuticals, and Takeda. Dr. Eugster is a clinical trial investigator for AbbVie. Dr. Mauras has received Institutional Research Support from Abbvie and is a consultant for Tolmar Pharmaceuticals.
